# Relationship between the risk of intestinal mucosal Epstein–Barr virus and/or cytomegalovirus infection and peripheral blood NK cells numbers in patients with ulcerative colitis: a cross-sectional study in Chinese population

**DOI:** 10.3389/fmicb.2024.1498483

**Published:** 2024-12-04

**Authors:** Ye Tian, Jinghua Dai, Yunfeng Yang, Xiaofeng Guo, Wei Wang, Fengxia Li, Juzi Wang, Ruiyun Liu

**Affiliations:** 1 Department of Gastroenterology, Shanxi Provincial People’s Hospital Affiliated to Shanxi Medical University, National Clinical Research Center for Digestive Diseases, Shanxi Inflammatory Bowel Disease Center, Taiyuan, China; 2 School of Nursing, Shanxi Medical University, Shanxi Provincial People’s Hospital Affiliated to Shanxi Medical University, Taiyuan, China; 3 Department of Laboratory Medicine, Shanxi Provincial People’s Hospital Affiliated to Shanxi Medical University, Taiyuan, China; 4 Nursing Department, Shanxi Provincial People’s Hospital Affiliated to Shanxi Medical University, Taiyuan, China; 5 Shanxi Children’s Hospital Affiliated to Shanxi Medical University, Taiyuan, China

**Keywords:** ulcerative colitis, cytomegalovirus, Epstein–Barr virus, opportunistic infections, NK cells

## Abstract

**Objective:**

This study aimed to analyze the relationship between the risk of common opportunistic pathogens Epstein–Barr virus (EBV) and cytomegalovirus (CMV) infection in intestinal mucosal tissues of Ulcerative Colitis (UC) patients and the number of peripheral blood NK cells.

**Methods:**

UC patients admitted to a third-grade class-A hospital from January 2018 to December 2023 were selected as research population. Clinical data of the patients were collected from the electronic medical record system. Additionally, samples of intestinal mucosal tissues were obtained for real-time fluorescence quantitative PCR to detect and analyze the viral load of CMV and EBV. Blood samples were collected for lymphocyte subsets analysis. Multivariable logistic regression models analyses was used to determine the odds ratio (OR) and 95% confidence interval (95% CI) for the independent association between NK cells and EBV/CMV infections in UC. We further applied the restricted cubic spline analysis and smooth curve fitting to examine the non-linear relationship between them.

**Results:**

378 UC patients were enrolled. Of these patients, there were 194 patients (51.32%) with EBV /CMV infection. In multivariable logistic regression analyses NK cells was independently associated with EBV and/or CMV infection after adjusted potential confounders (OR 8.24, 95% CI 3.75–18.13, *p* < 0.001). A nonlinear relationship was found between NK cells and EBV/CMV infections, which had a threshold around 10.169. The effect sizes and CIs below and above the threshold were 0.535 (0.413–0.692), *p* < 0.001 and 1.034 (0.904–1.183), *p* > 0.05, respectively.

**Conclusion:**

There was a non-linear relationship between NK cells and EBV/CMV infections. The risk for EBV/CMV infections was not increased with increasing NK cells in individuals with NK cells ≥ 10.169%, whereas the risk for EBV and/or CMV infection was increased with an decreasing NK cells in those with NK cells < 10.169%. The risk of EBV/CMV infections increases when NK cells were below a certain level.

## 1 Introduction

Since the end of the 20th century, the incidence and prevalence of inflammatory bowel disease (IBD) have increased in Asian countries, and China is one of the regions with the highest incidence of IBD in Asia (Ran et al., 2021). UC is a refractory, chronic, non-specific inflammatory response disease of the bowel, and is characterized by alternating exacerbations and remissions (Zhang et al., 2022). The main therapeutic drugs such as glucocorticoids, immunosuppressive agents, and biological agents have immunosuppressive effects (Ungaro et al., 2017). The immune function of most patients is suppressed for a long time due to the disease itself and the action of therapeutic drugs, resulting in an increased risk of opportunistic infections (Ljungman et al., 2002). The most common pathogens of opportunistic infections in UC populations include EBV and CMV (Groves et al., 2021; Kerr, 2019; Albatati et al., 2020). EBV, a member of the herpes virus family, has been reported to infect more than 90% of people in childhood (Zhang et al., 2022). Because they co-evolved with humans, both viruses have developed a unique ability to establish lifelong infections (McGeoch et al., 2000).

In fact, after initial infection, they lie dormant in specific cell populations, namely memory B cells of EBV (Chiu and Sugden, 2016) and monocytes/bone marrow/endothelial cells precursors of CMV (Jarvis and Nelson, 2002). That is, latent infection, it is defined as a state in which the viral genome is preserved, but the viral gene expression is highly restricted, and the viral replication process does not occur (Ciccocioppo et al., 2021). The reversibility of latent infection largely depends on t cell-mediated immune control and changes in the differentiation/ activation state of latent virus-infected cells (Murata and Tsurumi, 2014; Jarvis and Nelson, 2002). Reactivation of latent EBV/CMV infection in UC patients can complicate the condition and may manifest hormonal nonresponse or hormonal resistance. A large amount of evidence shows that viral reactivation is the main culprit leading to the development of the disease, which is easy to develop into acute and severe UC, and significantly increases the incidence of complications such as colectomy (Papadakis et al., 2001; Kishore et al., 2004; Domènech et al., 2008; Maher and Nassar, 2009; Núñez Ortiz et al., 2022) and mortality (Kishore et al., 2004; Gauss et al., 2015). Therefore, antiviral therapy is required after CMV and/or EBV reactivation in UC patients, and treatment can reduce hormone resistance and increase the efficacy of immunosuppressant use.

However, some studies (Huang et al., 2024; Pillet et al., 2016; Hsieh et al., 2024) still show that antiviral therapy is controversial in reducing the risk of serious complications such as colectomy, which may be related to the immune status of patients. A variety of immune factors in the body can reflect immune function to a certain extent. NK cells are activated after viral infection and play a role in the early stages before inducing the T cell response and in the latency period after the T cell response is established. Depletion of NK cells and T cells has been shown to lead to EBV/CMV reactivation, suggesting that these two lymphocyte subpopulations are critical for suppressing infection (Vieira Braga et al., 2015). Moreover, early studies have shown that natural killer cells play a major role in controlling CMV infection in the early and latent stages of infection (Kuijpers et al., 2008). At present, whether the number of NK cells in a certain immune state is an important factor affecting the increased risk of opportunistic infection in UC patients, and the quantification relationship remains to be verified. Therefore, early inference of the risk of viral reactivation through quantified NK cells analysis in UC patients, which is crucial to explore the pathogenesis of common opportunistic infections associated with UC and delay the development of adverse prognosis.

## 2 Materials and methods

### 2.1 Study population

**Figure 1:**
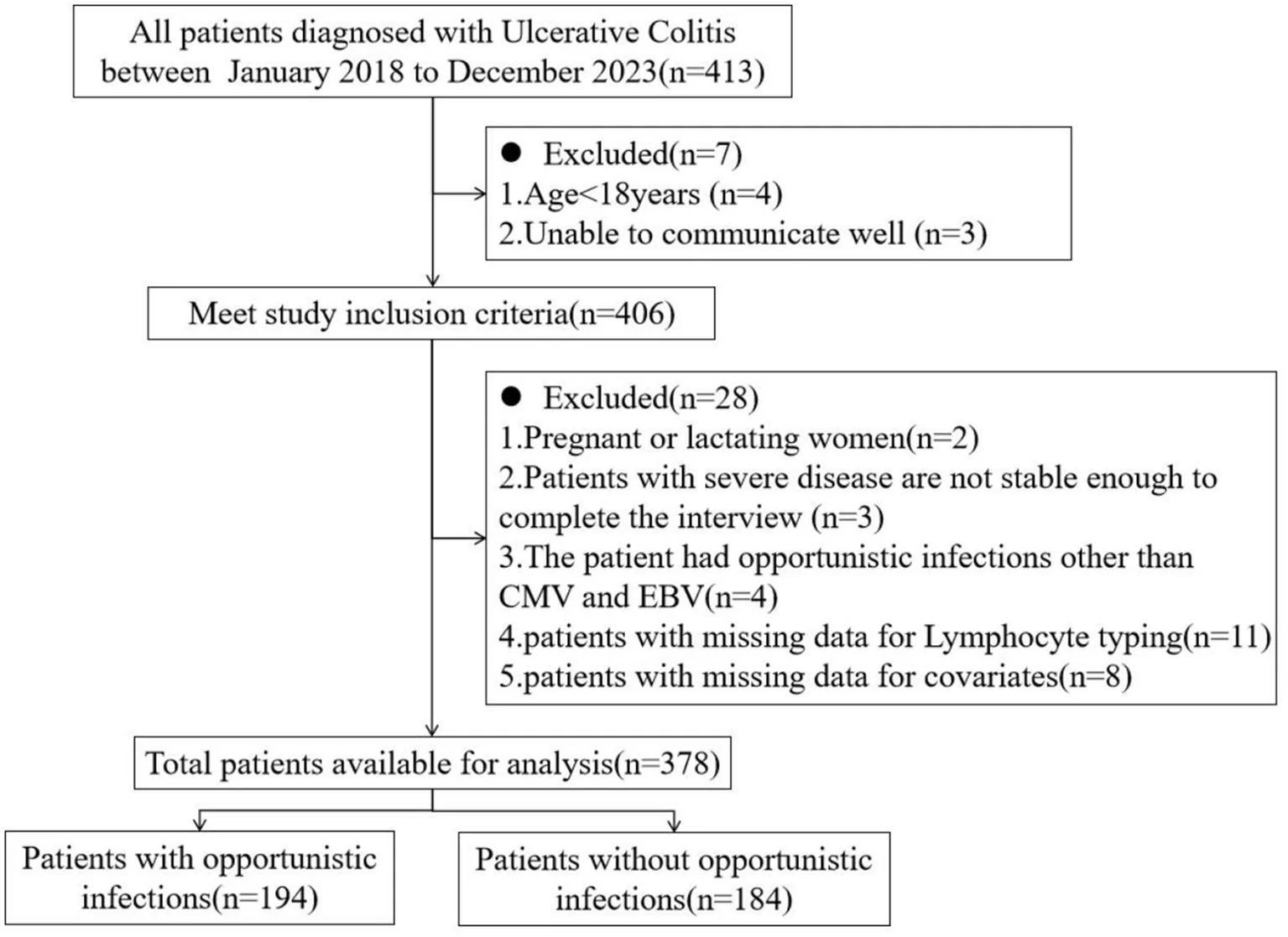
Flow diagram of inclusion and exclusion.

In this study, a cross-section of UC patients admitted to a third-grade class-A hospital from January 2018 to December 2023 were selected as research population. The inclusion criteria were as following: All patients met the diagnostic criteria for UC in the Guidelines for the Diagnosis and Treatment of Adult Ulcerative Colitis issued by PANCCO in July 2022 (Juliao-Baños et al., 2022); age > 18 years old; be able to communicate well and be willing to accept follow up. Establish a follow-up file for each patient. Exclusion criteria: pregnant or lactating women; Unstable vital signs and inability to complete the interview; Patients with immunodeficiency disease, malignant tumor; Patients with acute and chronic infectious enteritis, ischemic colitis, toxic megacolon, intestinal perforation and other serious diseases; Patients with a history of total (secondary) intestinal resection; The patient’s clinical data were incomplete; Patients with opportunistic infections other than CMV and EBV were excluded because there were fewer than 5 cases in the population. The inclusion and exclusion of patients in this study were shown in Figure 1. This study was approved by the Ethics Committee of the hospital (approval number: 2022SYKLSZD442), and all patients signed informed consent.

### 2.2 Methods

#### 2.2.1 Clinical data collection and activity score

Follow-up files of patients were established, and clinical data of the patients were collected from the electronic medical record system, including sex, age, course of disease, disease activity, lesion scope, routine physical examination data, laboratory examination, imaging examination, endoscopy, histological and pathological examination results, drug treatment history, nutritional status, etc. The improved Mayo score system (Xing et al., 2021) was used to evaluate the UC activity of hospitalized patients, and UC patients were divided into remission stage (Mayo score ≤ 2 and no single score > 1) and active stage (Mayo score > 2) according to the comprehensive results of patients’ symptoms and signs, relevant laboratory results, endoscopic findings and overall physician evaluation. The Mayo scores of 3 to 5 were classified as mildly active, 6 to 10 as moderately active, and 11 to 12 as severely active. According to the results of UC activity assessment, UC patients in remission were excluded from the study. Therefore, the number of such patients was very small, and the analysis results were not enough to represent the situation of this group. Montreal classification was used to evaluate the extent of the lesions, with E1 being rectal type, E2 left half colon type, and E3 extensive colon type (Satsangi et al., 2006).

#### 2.2.2 The patients’ intestinal mucosal tissue samples were taken for quantitative real-time PCR detection of CMV and EBV viral load analysis

Quantitative Real-time PCR detection of colon mucosal tissue is the gold standard in the diagnosis of UC combined with CMV/EBV infection, which is helpful for correct diagnosis and treatment (Ciccocioppo et al., 2015; Jena et al., 2022). It has been reported that the sensitivity and specificity of CMV DNA detected by qPCR in colon mucosal tissue are 92.0–96.7% and 93.0–98.7% (Groves et al., 2021). During the endoscopic examination, the patient was under anesthesia, and the doctor inserted an endoscope through the anus to observe the intestinal cavity through a monitor. Then located the appropriate site to obtain tissue specimens for colonic mucosal lesions. Gently scraped 2–3 small pieces of mucosal tissue from the surface of the colon using a biopsy forceps and placed them in a clean EP tube. When sampling, attention should be paid to sampling in the granulation tissue around ulcers or deep ulcer. The samples were immediately sent for examination after being frozen in liquid nitrogen to ensure the timeliness of the test results. Inspectors used EBV PCR fluorescence assay quantitative diagnostic kit (Sanure Biotech Inc. Hunan, China) and CMV PCR fluorescence quantitative diagnostic kit (Sanure Biotech Inc. Hunan, China) and Bio-Rad CFX-96 real-time fluorescence quantitative PCR. The reaction volume was 50 μL, including 37 μL reaction liquid, 2 μL enzyme mixture, 1 μL internal standard and 10 μL DNA extract. PCR reaction conditions were: 50°C for 2 min, 94°C for 5 min, then 45 cycles at 94°C for 15 s and 57°C for 30 s. The tissue results were expressed as the copy number of the viral genome per mg of tissue (Wang et al., 2022). Through the study of reference values, it was determined that the Ct positive judgment value of the target gene detected by this kit was 39, and the samples whose Ct value was less than 39 were reported as positive DNA of Epstein–Barr virus or CMV virus.

#### 2.2.3 Blood samples of patients were collected for lymphocyte subsets analysis

In the early morning, 5 mL of peripheral venous blood was extracted from all enrolled patients on an empty stomach, and red blood cell lysis was performed to prepare mononuclear cell suspension. Select specific antibodies and fluorescent dyes for labeling (matching reagents from BD Company of the United States, Lymphocyte subsets detection kit- BD MultiTEST IMK Kit). It comprises two 4-color panels, each panel in one tube, there are two reagents (CD3 FITC /CD8 PE / CD45 Per CP / CD4 APC and CD3 FITC / CD16 PE + CD56 PE / CD45 PerCP /CD19 APC). Briefly, to stain a sample, 50 μL whole blood was dispensed into a specimen ID labeled Trucount tube, 20 μL respective reagent was added, and the tube was vortexed and incubated for 15 min in the dark. At the end of the incubation time, 450 μL of a BD proprietary buffered lysing solution containing <15% formaldehyde and < 50% diethylene glycol (Lysis Buffer, 10X, BD Bio-sciences, San Jose, CA) was added to each tube, and tubes were incubated again in the dark for 15 min, the labeled cells were fixed and washed; The types and numbers of lymphocyte subsets, including total T lymphocytes (CD3+), total B lymphocytes (CD3-CD19+) and NK cells (CD3-CD16 + CD56+), were measured by BD FACSAria2 flow cytometry, all expressed as percentages.

#### 2.2.4 Statistical analysis

Patient characteristics were analyzed using quartile stratification of NK cell numbers. The quartile stratification of the number of NK cells was conducted by grouping continuous variables. Calculated the values of P25, P50, and P75. According to these values, the data were divided into four groups. Categorical variables are expressed as numbers and percentages. Continuous variables are represented by mean and standard deviation (SD) for normal distributions and median and interquartile (IQR) for skewered distributions. We used Chi-square tests, one-way ANOVA and Kruskal-Wallis tests, respectively, to compare categorical, normally distributed and non-normally distributed continuous variables. Further *post-hoc* analysis, for categorical variables, Chi-square test was used, and Bonferroni *x*^2^ segmentation method with adjusted test level was used on the basis of Chi-squared test to conduct multiple comparisons among multiple sample rates. The test level *α*’ was 0.0083 (0.05/6groups). Severity of disease is an ordered categorical variable. We used multiple comparison of rates by Kruskal-Walli rank sum test. The measurement data were analyzed using multiple comparison LSD-t test among multiple sample means based on ANOVA.

To investigate the relationship between the number of NK cells and the incidence of EBV and/or CMV infection in UC patients, Logistic univariate linear regression analysis and multivariate regression analysis were performed to determine the odds ratio (OR) and 95% confidence interval (95% CI). Among them, univariate Logistic regression analysis ‌ was used to evaluate the magnitude and direction of the influence of a single factor on the outcome variable. And then variables that had statistically significant on the occurrence of EBV and/or CMV infection were retained. Then we selected the variables with the NK cells’ adjusted effect size (*β* or OR) change greater than or equal to 10%, combined with clinical experience and previous studies (Li Wai Suen et al., 2024; Li Wai Suen et al., 2020) to determine that the adjusted variables included: age, clinical classification, extent of disease, severity of disease, nutritional status - indicator albumin level, medication history - use of corticosteroids or immunosuppressants. The selection process of the adjusted variables and the related regression formulas were detailed in Supplementary Table S1. These confounding factors were controlled for further multivariate regression analysis.

According to the recommendation of the STrengthening the Reporting of OBservational studies in Epidemiology (STROBE) statement (Vandenbroucke et al., 2007), analyses were first performed without adjustment. Further analysis was carried out by cumulative adjustment variables. So the stability of the relationship was determined by using unadjusted models and multi-factor adjusted models according to continuous variables (calculated as OR/HR per 5% NK cells’ proportion reduction) and categorical variables (quartiles), respectively. Analysis was performed in a crude model (Model 1: NK as a continuous variable adjusting variable age), and further cumulative adjustments were made for clinical classification (model 2), lesion extent and disease severity (model 3), nutritional status-indicator albumin level (model 4), and medication history (use of glucocorticoids or immunosuppressants) of the analysis variables.

To examine the non-linear relationship between the number of NK cells and the incidence of EBV and/or CMV infection, we used restricted cubic spline analysis in the Logistic regression model (Kong et al., 2018). Restricted cubic spline (RCS) regression was performed with 4 predefined knots at the 5th, 35th, 65th, and 95th percentile of NK cells to assess nonlinearity and examine the dose–response curve between the number of NK cells and the incidence of EBV and/or CMV infection after adjusting variables in Model 5. Spline regression fits smooth polynomial functions between predefined knots on a graph and joins them in a piecewise manner. Splines could be used to produce a non-linear model between a continuous prognostic variable and an outcome. If a nonlinear correlation was observed, a two-piecewise linear regression model was conducted to calculate the threshold effect of the number of NK cells on the incidence of EBV and/or CMV infection in terms of the smoothing plot/. When the number of NK cells and the incidence of EBV and/or CMV infection were evident in the smoothed curve, the recursive method automatically calculated the threshold, The likelihood-ratio test and the bootstrap resampling method were used to determine inflection points (Yu et al., 2018; Park et al., 2017; Liu et al., 2022).

We performed subgroup analyses for key characteristics that might modify the association between the number of NK cells and EBV and/or CMV infection assessed the potential confounding effects of sex, age, course of disease, clinical type, lesion extent, and disease severity on the number of NK cells. In the process of selecting subgroup variables, the variables with uneven distribution of subgroup sample size are excluded to reduce the deviation of results (Variables with too small sample size after grouping: Complication:Electrolyte imbalance, Complication:Pulmonary disease, Complication:Thyroid disease, Nutrition support:Human Albumin Solution, Medication history:Glucocorticoids OR immuno-suppressant). This study takes into account the sex variable as a grouping variable, which have a balanced sample size after stratification. *p* < 0.05 was considered significant. Multivariate logistic regression was used to assess the heterogeneity among subgroups, and likelihood ratio tests were used to examine the interaction between the number of NK cells and the incidence of EBV and/or CMV infection in each subgroup. If P for interaction<0.05, the relationship between the number of NK cells and the occurrence of EBV and/or CMV infection in the subgroup was statistically significant. If P for interaction>0.05, there was no statistical difference in the relationship between the two in the subgroup. The OR value and 95%CI measure the extent to which factors influence the outcome.

Statistical analysis was performed using R software (version4.2.1: The R Foundation for Statistical Computing, Vienna, Austria)^1^ and the Free Statistics software (version 1.9.2: Beijing Free Clinical Medical Technology Co. Ltd., Beijing, China). *p* values less than 0.05 (two-sided) were considered statistically significant. The formulas that were used for all the regression analyses were detailed in the Supplementary material.

## 3 Results

### 3.1 EBV and/or CMV infection in UC patients

The basic situation of ulcerative colitis patients with EBV and/or CMV infection is shown in Table 1. Of these patients, 184 patients did not have common EBV and/or CMV infection. There were 194 patients (51.32%) with common opportunistic bacterial infection CMV and/or EBV infection, of which, the number of people with one infection is 126 (64.95%). The number of people with two infections is 68 (35.05%) 0.204 were male and 174 were female, 278 patients over 40 years old (73.5%). The number of NK cells in patients with two common opportunistic bacterial infections was significantly lower than in other patients (*p* < 0.001).

**Table 1:** Common opportunistic infections in UC patients.

Demographic characteristics	Total (*n* = 378)	Types of Infection				*p*	F/x^2^
		CMV(−) EBV(−) (*n* = 184)	CMV(−) EBV(+) (*n* = 114)	CMV(+) EBV(−) (*n* = 12)	CMV(+) EBV(+) (*n* = 68)		
**Sex, n (%)**						0.443	2.686
Female	174 (46.0)	91 (49.5)	51 (44.7)	6 (50)	26 (38.2)		
Male	204 (54.0)	93 (50.5)	63 (55.3)	6 (50)	42 (61.8)		
**Age, n (%)**						0.088	Fisher
≤40 years	100 (26.5)	41 (22.3)	30 (26.3)	3 (25)	26 (38.2)		
>40 years	278 (73.5)	143 (77.7)	84 (73.7)	9 (75)	42 (61.8)		
**Course of disease, n (%)**						0.003	Fisher
<1 year	128 (33.9)	77 (41.8)	32 (28.1)	5 (41.7)	14 (20.6)		
1–3 years	129 (34.1)	57 (31)	35 (30.7)	2 (16.7)	35 (51.5)		
>3 years	121 (32.0)	50 (27.2)	47 (41.2)	5 (41.7)	19 (27.9)		
**Clinical type, n (%)**						<0.001	35.303
First episode type1	171 (45.2)	107 (58.2)	49 (43)	2 (16.7)	13 (19.1)		
Chronic relapsing type2	207 (54.8)	77 (41.8)	65 (57)	10 (83.3)	55 (80.9)		
**Lesion range: Montreal typing, n (%)**						<0.001	Fisher
E1	130 (34.4)	91 (49.5)	30 (26.3)	1 (8.3)	8 (11.8)		
E2	79 (20.9)	38 (20.7)	22 (19.3)	2 (16.7)	17 (25)		
E3	169 (44.7)	55 (29.9)	62 (54.4)	9 (75)	43 (63.2)		
**Severity of disease, n (%)**						<0.001	Fisher
Remission	167 (44.2)	122 (66.3)	35 (30.7)	2 (16.7)	8 (11.8)		
Medium	94 (24.9)	35 (19)	33 (28.9)	4 (33.3)	22 (32.4)		
Severe	117 (31.0)	27 (14.7)	46 (40.4)	6 (50)	38 (55.9)		
NK cells, Median (IQR)	9.8 (7.8, 13.8)	10.8 (9.1, 14.9)	9.7 (7.8, 13.7)	9.2 (7.1, 15.2)	6.4 (3.8, 8.8)	< 0.001	58.328

### 3.2 Baseline characteristics of study patients

Table 2 illustrates the baseline characteristics of all patients according to their NK cells stratified quartiles. The average age of the study participants was (49.5 ± 14.5) years. The post-hoc analysis (Table 3) showed that people with fewer NK cells tend to be the clinical type was chronic relapsing type; the lesion were classified as E1 or E3 in Montreal; the severity of the disease was severe, low ALB; the history of glucocorticoids or immunosuppressants; human albumin or nutritional support therapy was required.

**Table 2:** Patient characteristics and outcome parameters.

Patient characteristics	Total (*n* = 378)	NK cells stratified quartile				*P*	*F/x^2^*
		Q1 (*n* = 95) (0.8–7.8)	Q2 (*n* = 93) (7.9–9.7)	Q3 (*n* = 93) (9.8–13.7)	Q4 (*n* = 97) (13.8–32.8)		
**Sex, n (%)**						0.941	0.395
Female	174 (46.0)	43 (45.3)	43 (46.2)	41 (44.1)	47 (48.5)		
Male	204 (54.0)	52 (54.7)	50 (53.8)	52 (55.9)	50 (51.5)		
Age, Mean ± SD	49.5 ± 14.5	47.3 ± 15.2	51.6 ± 13.9	49.1 ± 14.3	50.0 ± 14.3	0.235	1.425
**Course of disease, n (%)**						0.506	5.302
<1 year	128 (33.9)	31 (32.6)	36 (38.7)	31 (33.3)	30 (30.9)		
1–3 years	129 (34.1)	37 (38.9)	23 (24.7)	33 (35.5)	36 (37.1)		
>3 years	121 (32.0)	27 (28.4)	34 (36.6)	29 (31.2)	31 (32)		
**Complication: high blood pressure, n (%)**						0.534	2.188
No	332 (87.8)	86 (90.5)	78 (83.9)	83 (89.2)	85 (87.6)		
Yes	46 (12.2)	9 (9.5)	15 (16.1)	10 (10.8)	12 (12.4)		
**Complication: electrolyte imbalance, n (%)**						0.248	4.13
No	342 (90.5)	85 (89.5)	83 (89.2)	89 (95.7)	85 (87.6)		
Yes	36 (9.5)	10 (10.5)	10 (10.8)	4 (4.3)	12 (12.4)		
**Complication: diabetes, n (%)**						0.652	Fisher
No	365 (96.6)	92 (96.8)	88 (94.6)	90 (96.8)	95 (97.9)		
Yes	13 (3.4)	3 (3.2)	5 (5.4)	3 (3.2)	2 (2.1)		
**Complication: pulmonary disease, n (%)**						0.523	2.247
No	294 (77.8)	71 (74.7)	73 (78.5)	77 (82.8)	73 (75.3)		
Yes	84 (22.2)	24 (25.3)	20 (21.5)	16 (17.2)	24 (24.7)		
**Complication: Cerebrovascular disease, n (%)**						0.976	Fisher
No	368 (97.4)	93 (97.9)	90 (96.8)	91 (97.8)	94 (96.9)		
Yes	10 (2.6)	2 (2.1)	3 (3.2)	2 (2.2)	3 (3.1)		
**Complication: heart disease, n (%)**						0.064	Fisher
No	362 (95.8)	89 (93.7)	86 (92.5)	91 (97.8)	96 (99)		
Yes	16 (4.2)	6 (6.3)	7 (7.5)	2 (2.2)	1 (1)		
**Complication: thyroid disease, n (%)**						0.695	1.447
No	323 (85.4)	78 (82.1)	79 (84.9)	81 (87.1)	85 (87.6)		
Yes	55 (14.6)	17 (17.9)	14 (15.1)	12 (12.9)	12 (12.4)		
**Clinical type, n (%)**						0.045	8.037
First episode type1	171 (45.2)	32 (33.7)	46 (49.5)	49 (52.7)	44 (45.4)		
Chronic relapsing type2	207 (54.8)	63 (66.3)	47 (50.5)	44 (47.3)	53 (54.6)		
**Lesion range: Montreal typing, n (%)**						< 0.001	21.564
E1	130 (34.4)	21 (22.1)	28 (30.1)	48 (51.6)	33 (34)		
E2	79 (20.9)	25 (26.3)	26 (28)	10 (10.8)	18 (18.6)		
E3	169 (44.7)	49 (51.6)	39 (41.9)	35 (37.6)	46 (47.4)		
**Severity of disease, n (%)**						< 0.001	27.075
Remission	167 (44.2)	28 (29.5)	40 (43)	60 (64.5)	39 (40.2)		
Medium	94 (24.9)	26 (27.4)	24 (25.8)	14 (15.1)	30 (30.9)		
Severe	117 (31.0)	41 (43.2)	29 (31.2)	19 (20.4)	28 (28.9)		
**Extra intestinal manifestations: Joint damage, n (%)**						0.576	Fisher
No	369 (97.6)	91 (95.8)	91 (97.8)	91 (97.8)	96 (99)		
Yes	9 (2.4)	4 (4.2)	2 (2.2)	2 (2.2)	1 (1)		
**Extra intestinal manifestations: skin and mucosa reactions, n (%)**						0.876	0.689
No	343 (90.7)	85 (89.5)	85 (91.4)	86 (92.5)	87 (89.7)		
Yes	35 (9.3)	10 (10.5)	8 (8.6)	7 (7.5)	10 (10.3)		
**Extra intestinal manifestations: Ocular lesion, n (%)**						0.12	Fisher
No	371 (98.1)	93 (97.9)	93 (100)	89 (95.7)	96 (99)		
Yes	7 (1.9)	2 (2.1)	0 (0)	4 (4.3)	1 (1)		
**Extra intestinal manifestations: Hepatobiliary disease, n (%)**						0.152	5.286
No	348 (92.1)	90 (94.7)	82 (88.2)	89 (95.7)	87 (89.7)		
Yes	30 (7.9)	5 (5.3)	11 (11.8)	4 (4.3)	10 (10.3)		
ALB, Mean ± SD	35.9 ± 6.8	34.3 ± 7.1	35.9 ± 6.3	36.7 ± 7.1	36.7 ± 6.5	0.049	2.645
HGB, Mean ± SD	120.5 ± 25.1	115.7 ± 25.0	123.6 ± 22.2	122.5 ± 28.5	120.2 ± 23.8	0.138	1.847
**Medication.history: Glucocorticoids OR Immunosuppressants, n (%)**						0.01	11.373
No	316 (83.6)	69 (72.6)	80 (86)	81 (87.1)	86 (88.7)		
Yes	62 (16.4)	26 (27.4)	13 (14)	12 (12.9)	11 (11.3)		
**Medication history: Glucocorticoids and Immunosuppressants, n (%)**						0.873	Fisher
No	364 (96.3)	91 (95.8)	89 (95.7)	91 (97.8)	93 (95.9)		
Yes	14 (3.7)	4 (4.2)	4 (4.3)	2 (2.2)	4 (4.1)		
**Medication history: Biological agents, n (%)**						0.655	Fisher
No	370 (97.9)	92 (96.8)	92 (98.9)	92 (98.9)	94 (96.9)		
Yes	8 (2.1)	3 (3.2)	1 (1.1)	1 (1.1)	3 (3.1)		
**Nutrition support: Enteral nutrition, n (%)**						0.048	7.918
No	310 (82.0)	70 (73.7)	76 (81.7)	83 (89.2)	81 (83.5)		
Yes	68 (18.0)	25 (26.3)	17 (18.3)	10 (10.8)	16 (16.5)		
**Nutrition support: Human Albumin Solution, n (%)**						0.038	8.445
No	321 (84.9)	72 (75.8)	83 (89.2)	81 (87.1)	85 (87.6)		
Yes	57 (15.1)	23 (24.2)	10 (10.8)	12 (12.9)	12 (12.4)		

ALB, albumin; HGB, hemoglobin.

**Table 3:** Multiple comparisons among NK cells stratified quartile groups.

Patient characteristics	NK cells stratified quartile groups											
	Q1 vs. Q2		Q1 vs. Q3		Q1 vs. Q4		Q2 vs. Q3		Q2 vs. Q4		Q3 vs. Q4	
	Value	*p*	Value	*p*	Value	*p*	Value	*p*	Value	*p*	Value	*p*
Clinical type, n (%)												
First episode type1	4.593	0.040	7.270	0.007	2.736	0.098	0.356	0.551	0.251	0.617	1.187	0.276
Chronic relapsing type2												
Lesion range: Montreal typing, n (%)												
E1	2.513	0.292	18.346	<0.001	3.881	0.144	10.784	0.005	2.011	0.366	5.971	0.051
E2												
E3												
Severity of disease, n (%)^a^												
Remission	1.950	0.307	4.852	<0.001	1.978	0.287	2.942	0.020	0.024	1.000	−2.926	0.021
Medium												
Severe												
ALB, Mean ± SD^b^	−1.541	0.116	−2.502	0.012	−2.370	0.016	−0.962	0.991	−0.829	0.394	0.133	0.894
Medication.history: Glucocorticoids OR Immunosuppressants, n (%)												
No	5.618	0.020	5.578	0.018	7.926	0.005	0.002	0.967	0.215	0.643	0.172	0.678
Yes												
Nutrition support: Enteral nutrition, n (%)												
No	1.567	0.229	8.202	0.004	2.756	0.097	2.866	0.090	0.169	0.681	1.700	0.192
Yes												
Nutrition support: Human Albumin Solution, n (%)												
No	6.357	0.013	3.565	0.059	4.513	0.034	0.379	0.538	0.182	0.669	0.039	0.844
Yes												

a is the multiple comparison Kruskal-Walli rank sum test of rates.

b is the multiple comparison LSD-t test of the mean.

### 3.3 Univariate analysis of risk factor associated with EBV and/or CMV infection in UC patients

Univariate analysis of risk factor associated with EBV and/or CMV infection in UC patients, reported as ORs and 95% CIs for the risk of that among patients with UC was summarized in Table 4. Course of disease, complication:electrolyte imbalance, pulmonary disease, thyroid disease, Clinical type, Lesion range, severity of disease, ALB, HGB, Medication history:glucocorticoids or immunosuppressants, nutrition support:human albumin solution, NK cells were significantly associated with EBV and/or CMV infection (*p* < 0.01). Other factors were not significantly associated with that.

**Table 4:** Univariate analysis of risk factor associated with EBV and/or CMV infection in UC patients.

Variables	OR (95%CI)	*p-*value
Sex		
Male	Ref.	
Female	1.31 (0.87–1.96)	0.194
Age	0.99 (0.98–1.01)	0.270
Course of disease		
<1 year	Ref.	
1–3 years	1.91 (1.16–3.13)	0.011
>3 years	2.14 (1.29–3.56)	0.003
Complication: High blood pressure		
No	Ref.	
Yes	1.27 (0.68–2.36)	0.452
Complication: Electrolyte imbalance		
No	Ref.	
Yes	2.69 (1.26–5.76)	0.011
Complication: Diabetes		
No	Ref.	
Yes	1.11 (0.37–3.37)	0.853
Complication: Pulmonary disease		
No	Ref.	
Yes	2.59 (1.55–4.34)	<0.001
Complication: Cerebrovascular disease		
No	Ref.	
Yes	1.44 (0.4–5.17)	0.58
Complication: Heart disease		
No	Ref.	
Yes	2.97 (0.94–9.37)	0.064
Complication: Thyroid disease		
No	Ref.	
Yes	2.39 (1.3–4.41)	0.005
Clinical type		
First episode type1	Ref.	
Chronic relapsing type2	2.82 (1.86–4.29)	<0.001
Lesion range		
E1	Ref.	
E2	2.52 (1.41–4.49)	0.002
E3	4.84 (2.95–7.93)	<0.001
Severity of disease		
Remission	Ref.	
Medium	4.57 (2.66–7.84)	<0.001
Severe	9.04 (5.22–15.65)	<0.001
Extra intestinal manifestations: Joint damage		
No	Ref.	
Yes	1.19 (0.31–4.5)	0.797
Extra intestinal manifestations: Skin and mucosa reactions		
No	Ref.	
Yes	1.68 (0.82–3.45)	0.155
Extra intestinal manifestations: Ocular lesion		
No	Ref.	
Yes	1.27 (0.28–5.75)	0.756
Extra intestinal manifestations: Hepatobiliary disease		
No	Ref.	
Yes	1.26 (0.6–2.68)	0.542
ALB	0.92 (0.89–0.95)	<0.001
HGB	0.99 (0.98–1.00)	0.003
Medication history: Glucocorticoids OR immunosuppressants		
No	Ref.	
Yes	8.49 (3.91–18.42)	<0.001
Medication history: Glucocorticoids AND immunosuppressants		
No	Ref.	
Yes	3.63 (1.00–13.21)	0.051
Medication history: Biological agents		
No	Ref.	
Yes	2.9 (0.58–14.58)	0.195
Nutrition support: Enteral nutrition		
No	Ref.	
Yes	1.25 (0.74–2.12)	0.407
Nutrition support: Human Albumin Solution		
No	Ref.	
Yes	2.81 (1.52–5.22)	0.001
TOTEL B cells	1.02 (0.98–1.06)	0.441
TOTEL T cells	1.02 (0.99–1.05)	0.146
NK cells.	0.91 (0.87–0.95)	<0.001

ALB, albumin; HGB, hemoglobin; NK cells, natural killer cells.

### 3.4 Multivariate logistic regression analysis of NK cells on the EBV and/or CMV infection in UC

In multivariable logistic regression analyses with NK cells (%)-stratified quartile, there was a 5.80 times increased risk of EBV and/or CMV infection in the lowest NK cells (%) quartile compared to highest quartile (OR 8.24, 95% CI 3.75–18.13, *p* < 0.001), independent of potential confounders (Table 5, model 5). In multivariable logistic regression analyses, with adjustment for age, NK cells (%) expressed as a continuous variable was inversely associated with EBV and/or CMV infection [for per 5% decrease of NK cells (%): OR 1.60, 95% CI 1.28–2.01, *p* < 0.001; Table 2]. This association remained significant (OR 1.49 95% CI 1.16–1.93, *p* < 0.001), independent of potential confounders (Table 5, model 5). The statistical results were robust among all models.

**Table 5:** Multivariate logistic regression analysis of NK cells on the EBV and/or CMV infection in UC.

Model	NK cells (%)^a^		NK cells (%)-stratified quartile			
	(*n* = 378)		Q1 (*n* = 95)	Q2 (*n* = 93)	Q3 (*n* = 93)	Q4 (*n* = 97)
	OR (95%CI)	*P*	OR (95%CI)	OR (95%CI)	OR (95%CI)	Reference
Model1^b^	1.60 (1.28–2.01)	<0.001	7.86 (3.96–15.60)*	1.08 (0.61–1.93)	0.94 (0.52–1.68)	1.00
Model2^c^	1.58 (1.25–1.99)	<0.001	7.69 (3.82–15.49)*	1.13 (0.62–2.06)	1.01 (0.55–1.82)	1.00
Model3^d^	1.59 (1.25–2.03)	<0.001	9.57 (4.44–20.65)*	1.17 (0.61–2.23)	1.52 (0.77–2.97)	1.00
Model4^e^	1.55 (1.21–1.98)	<0.001	8.90 (4.12–19.24)*	1.15 (0.60–2.21)	1.49 (0.75–2.94)	1.00
Model5^f^	1.49 (1.16–1.93)	0.002	8.24 (3.75–18.13)*	1.10 (0.56–2.15)	1.42 (0.71–2.84)	1.00

Multivariable logistic regression to assess the association of NK cells’ proportion with incidence of EBV and/or CMV infection. *p for logistic regression analyses is < 0.001.

a NK cells’proportion was entered as a continuous variable per 5% decrease.

b Model 1: adjusted for age in continuous analyses, no adjustment for NK cells’ proportion-stratified quartile.

c Model 2: adjusted as for model 1, additionally adjusted for Clinical type.

d Model 3: adjusted as for model 2, additionally adjusted for Lesion range and Severity of disease.

e Model 4: adjusted as for model 3, additionally adjusted for ALB (Nutritional status index).

f Model 5: adjusted as for model 4, additionally adjusted for Medication history (glucocorticoids or immunosuppressants).

### 3.5 Nonlinear relationship analysis between NK cells and EBV and/or CMV infection in UC

**Figure 2:**
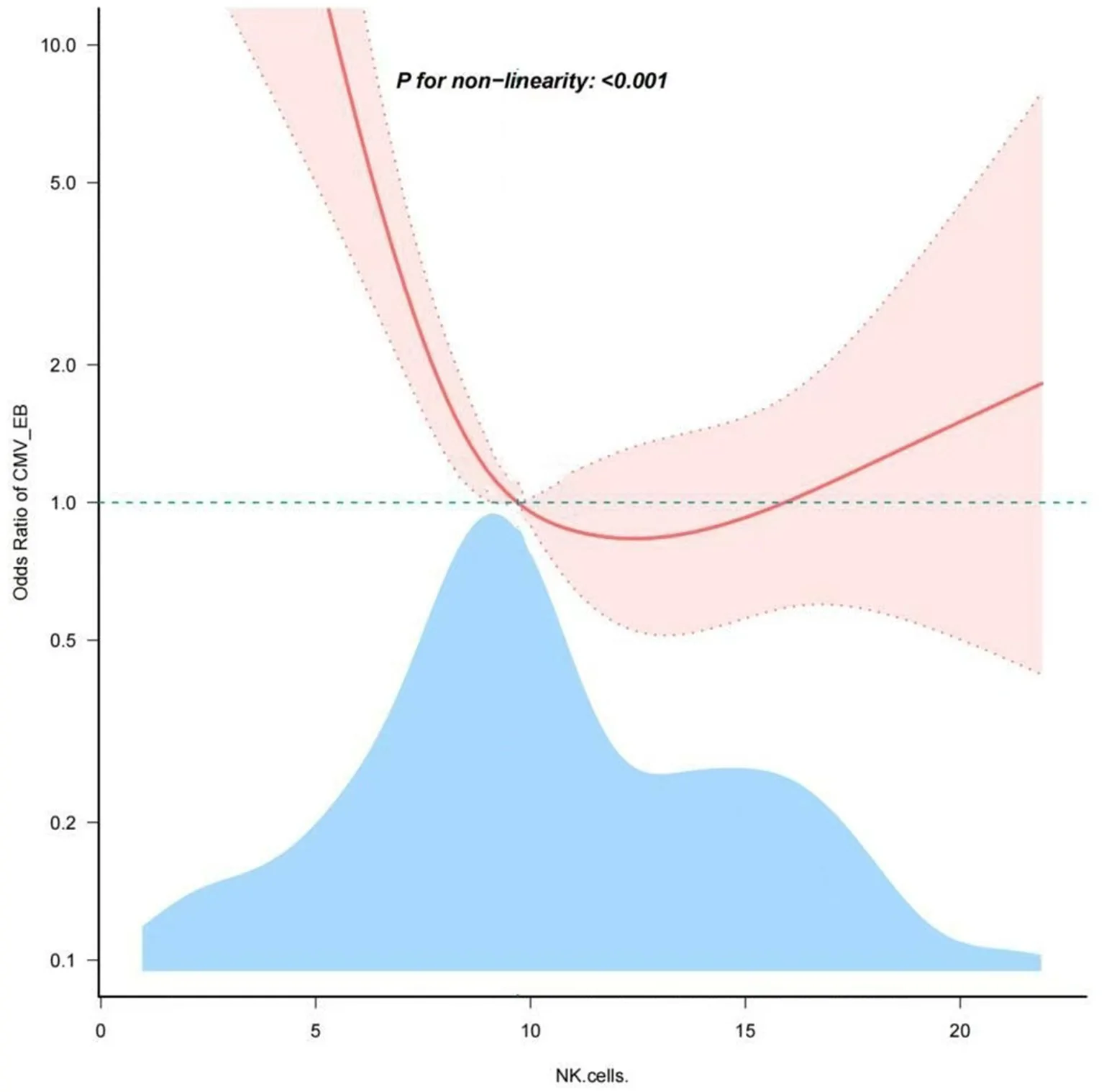
Nonlinear dose–response relationship of NK cells and the opportunistic infections. Adjustment factors included age, clinical type, lesion range, severity of disease, ALB and medication history. The red line and pink area represent the estimated values and their corresponding 95% confidence intervals, respectively.

Through restricted cubic spline model for smooth curve fitting, we found that the relationship between NK cells and EBV and/or CMV infection in UC was nonlinear (Figure 2), the *p* value for the likelihood ratio test was less than 0.001 (Table 6); therefore, we used a two-part model to fit the association between NK cells and EBV and/or CMV infection in UC. We found an inflection point at approximately 10.169%. On the left side of the inflection point, the effect size was 0.535 [OR: 0.535, 95% CI (0.413–0.692), *p* < 0.001], and on the right side of the inflection point, the effect size was 1.034 [OR: 1.034, 95% CI (0.904–1.183), *p* > 0.05]. This means that the risk of EBV and/or CMV infection was reduced by 46.5% with every 1% increase in NK cells. There was no association between NK cells and EBV and/or CMV infection when NK cells was ≥10.169% (Table 6). This means that the risk of EBV and/or CMV infection no longer decreases with increasing NK cells.

**Table 6:** Threshold effect analysis of the relationship of NK cells and the EBV and/or CMV infection.

Item	OR	95%CI	*p*-value
Inflection point (%)	10.169	(9.959, 10.379)	
NK cells (%) < 10.169%	0.535	(0.413–0.692)	<0.001
NK cells (%) ≥ 10.169%	1.034	(0.904–1.183)	0.6242
Likelihood Ratio test			<0.001
Non-linear Test*1			<0.001

Adjusted for age, Clinical type, Lesion range, Severity of disease, ALB and Medication history.

### 3.6 Subgroup analyses of factors influencing the association between NK cells and the EBV and/or CMV infection in UC

**Figure 3:**
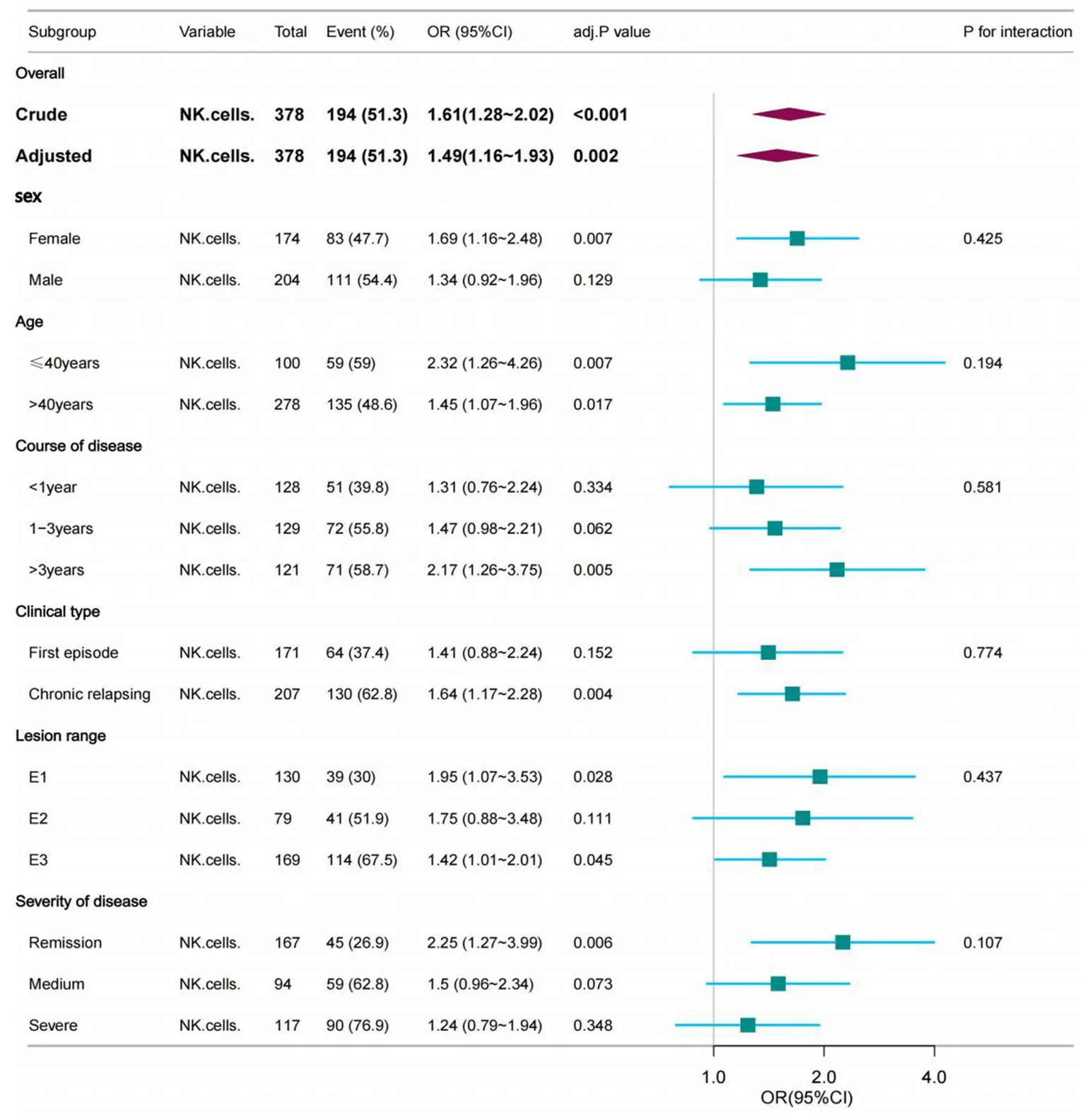
The association between NK cells and the opportunistic infections in subgroups. The *p*-value for interaction represents the likelihood of interaction between the variable and NK cells. Age was entered as a categorical variable. Adjusted for age, Clinical type, Lesion range, Severity of disease, ALB and Medication history.

To detect whether the association between NK cells number and incidence of EBV and/or CMV infection was stable in different subgroups, analyses and interactive analyses were stratified according to confounders. No significant interaction was observed in any subgroups (*p*-value for interaction>0.05). Nevertheless, in the female subgroup, with age ≤ 40 years, course of disease>3 years, clinical type was chronic relapsing, lesion range was E1 or E3 were associated with a greater risk for EBV and/or CMV infection compared with the corresponding subgroup. Subgroup analyses were adjusted for age, clinical type, lesion range, severity of disease, ALB and medication history (Table 7; Figure 3).

**Table 7:** The association between NK cells and the EBV and/or CMV infection in subgroups.

Subgroup	Variable^a^	n	n.event%	crude.OR95CI	crude.P	adj.OR95CI	adj.P	P for interaction
Sex^b^								
Female	NK cells	174	83 (47.7)	1.61 (1.15–2.26)	0.006	1.69 (1.16–2.48)	0.007	0.425
Male	NK cells	204	111 (54.4)	1.61 (1.19–2.19)	0.002	1.34 (0.92–1.96)	0.129	
Age^b^								
≤40 years	NK cells	100	59 (59)	1.95 (1.2–3.15)	0.007	2.32 (1.26–4.26)	0.007	0.194
>40 years	NK cells	278	135 (48.6)	1.5 (1.16–1.94)	0.002	1.45 (1.07–1.96)	0.017	
Course of disease^b^								
<1 year	NK cells	128	51 (39.8)	1.54 (1–2.37)	0.051	1.31 (0.76–2.24)	0.334	0.581
1-3 years	NK cells	129	72 (55.8)	1.47 (1.05–2.05)	0.023	1.47 (0.98–2.21)	0.062	
>3 years	NK cells	121	71 (58.7)	2.13 (1.33–3.42)	0.002	2.17 (1.26–3.75)	0.005	
Clinical type^c^								
First episode	NK cells	171	64 (37.4)	1.52 (1.03–2.25)	0.037	1.41 (0.88–2.24)	0.152	0.774
Chronic relapsing	NK cells	207	130 (62.8)	1.63 (1.23–2.18)	0.001	1.64 (1.17–2.28)	0.004	
Lesion range^d^								
E1	NK cells	130	39 (30)	2.18 (1.25–3.78)	0.006	1.95 (1.07–3.53)	0.028	0.437
E2	NK cells	79	41 (51.9)	1.67 (0.98–2.85)	0.059	1.75 (0.88–3.48)	0.111	
E3	NK cells	169	114 (67.5)	1.45 (1.09–1.94)	0.011	1.42 (1.01–2.01)	0.045	
Severity of disease^e^								
Remission	NK cells	167	45 (26.9)	2.38 (1.42–4.01)	0.001	2.25 (1.27–3.99)	0.006	0.107
Medium	NK cells	94	59 (62.8)	1.55 (1.02–2.34)	0.04	1.5 (0.96–2.34)	0.073	
Severe	NK cells	117	90 (76.9)	1.26 (0.88–1.82)	0.212	1.24 (0.79–1.94)	0.348	

a NK cells’ proportion was entered as a continuous variable per 5% decrease.

b Adjusted for age, Clinical type, Lesion range, Severity of disease, ALB and Medication history.

c Adjusted for age, Lesion range, Severity of disease, ALB and Medication history.

d Adjusted for age, Clinical type, Severity of disease, ALB and Medication history.

e Adjusted for age, Clinical type, Lesion range, ALB and Medication history.

## 4 Discussion

To maintain intestinal homeostasis, a balanced interplay between the intestinal microbiota, intestinal epithelial cells (IECs), and immune cells is required. In UC, homeostasis is disrupted and destruction of the epithelium, atrophy of crypts and immune cell infiltration is observed (Baumdick et al., 2023), opportunistic infections are very likely to occur. Despite opportunistic infection have attracted more and more attention in UC, several key issues remain unresolved, such as their role in UC, the possible risk factors and the effects on immune function. The research so far has only revealed that dysregulated responses of innate immune cells are observed and implicated in the pathogenesis of UC (Leppkes and Neurath, 2020). This study measured EBV and/or CMV viral loads in the intestinal mucosa to accurately diagnose patients with positive infections. The research shows that there were 51.32% of UC patients with common opportunistic infection CMV and/or EBV virus. The prevalence was slightly lower than previously reported study (Wang et al., 2022). However, the prevalence is higher for the population as a whole. From the perspective of immune cells, our study reveals the intrinsic relationship between the number of immune cells and opportunistic infection with viruses. Natural killer (NK) cells are innate lymphocytes that play an integral role in the host’s immune defense against pathogens. Some studies have pointed out that depletion of NK cells is linked with dramatic increases in colonic damage, leukocyte infiltration and pro-inflammatory profiles (Hall et al., 2013; Baumdick et al., 2023). It also suggests that there may be a certain relationship between the internal changes of NK cells and the occurrence and development of the disease in UC patients with EBV and/or CMV infection. In this cross-sectional study, we tried to examine the NK cells associated with EBV and/or CMV infection in UC. An non-linear L-shaped association between them was found in the study, with an inflection point of almost 10.169%. The correlations between NK cells and EBV and/or CMV infection of UC patients were totally different below and above the inflection point which was around 10.169%. The association remained robust in sensitivity and subgroup analyses.

In recent publication using mass cytometry on blood samples of UC patients reported that innate NK cells (CD3-CD16 + CD56+) were significant decreased in overall UC patients (Pappa et al., 2023). Another study found that the reduction of these potential regulatory immune subsets (innate NK-like T cells among others) was observed in UC, and did not associate with disease severity (Van Unen et al., 2022). Current studies have not focused on the trend of NK cells in UC patients with EBV and/or CMV infection. Compared with previous studies, the present study analyzed data from the last 5 years after adjusting for potential confounders using multivariable regression analysis to make the results generalizable to the UC adult population. Dose response analysis revealed a non-linear relationship between NK cells and EBV and/or CMV infection. In particular, the risk for EBV and/or CMV infection was not increased with increasing NK cells in individuals with NK cells≥10.169%, whereas the risk for EBV and/or CMV infection was increased with an decreasing NK cells in those with NK cells <10.169%. In other words, the risk of EBV and/or CMV infection increases only when NK cells are below a certain level. Additionally, the association between NK cells and EBV and/or CMV infection remained stable in multivariate adjustment of logistics regression analysis.

Previous studies have suggested potential biological mechanisms underlying the association between the NK cells and EBV and/or CMV infection in UC. NK cells have immunomodulatory properties *in vivo* during chronic inflammation and autoimmunity (Zitti and Bryceson, 2018; Poggi et al., 2019;Lees, 2015). NK cells do not express specific antigen recognition receptors, but recognize “self” and “non-self” through surface activated receptors and inhibitory receptors, and directly kill the target cells infected by the virus. When virus infection occurs, the expression of MHC class I molecules on the surface of virus-infected cells is deleted or down-regulated, while some non-MHC Class I molecular ligands that can be recognized by NK cells on the surface are abnormal or up-regulated, and NK cells are further activated to kill the target cells of virus infection by releasing perforin, granase, TNF-*α* and expressing FasL (Cao, 2017). The above are the recognition, activation and cytotoxic effects of NK cells on the target cells of virus infection under physiological state. Cytokine stimulation of NK cells of UC patients results in reduced activation of mTORC1 and impaired IFN-*γ* production (Pålsson-McDermott and O’Neill, 2020). However, additional well-designed longitudinal investigations are required to further evaluate the association between the NK cells and inflammatory potential of EBV and/or CMV infection. Understanding how NK cells behave in the peripheral blood of UC patients is important not only in terms of understanding cell defects in the course of disease, but may also inform new therapeutic approaches for these patients (O’Brien and Finlay, 2019).

NK cells are key immune cells in the fight against infection and cancer. They can directly kill pathogens as well as modulate the innate and adaptive immune response. But circulating NK cells of UC patients have an unbalanced metabolic profile, with faulty mitochondria and reduced capacity to kill. These aberrations in NK cell metabolism may contribute to defective killing and thus the secondary infections and increased risk of cancer observed in UC patients (Zaiatz Bittencourt et al., 2021). In this study, the correlation between the number of NK cells in peripheral blood of UC patients and the risk of EBV and/or CMV infection was analyzed. It can be seen that in the multivariate logistic regression analysis of NK cells (%) quartile stratified, compared with the highest quartile, after correcting for all potential confounding factors, Patients with the lowest quartile of NK cells (%) had a 7.24-fold increase in the risk of opportunistic infection (OR 8.24, 95% CI 3.75–18.13, *p* < 0.001), which fully revealed the widespread reduction in the number of peripheral NK cells in UC patients with EBV and/or CMV infection. Our study highlighted the urgent need to further explore immune defects of different cell types in UC patients and target to regulate immune cell function to clarify if this may be a future therapeutic way to treat UC with EBV and/or CMV infection patients.

Previous studies did not investigate the association and interaction between NK cells and EBV and/or CMV infection in different subgroups. Our subgroup analysis showed that female, age ≤ 40 years, course of disease>3 years, clinical type was chronic relapsing, lesion range was E1 or E3 had a higher risk of EBV and/or CMV. Previous studies (Altunal et al., 2023) have shown that long disease duration were significant risk factors for CMV colitis among patients with UC activation. This is consistent with our research. For the differences in disease susceptibility caused by sex, we can try to reveal them in subsequent studies on susceptibility genes. Owing to the limited sample size of participants with UC patients in our study, this result should be interpreted with caution, and more well-designed prospective studies in this field are warranted.

Further investigation on the functional role of NK cells in UC is necessary. NK cells from UC patients with increased viral loads should also be investigated based on the importance of NK cells in the elimination of pathogens. Our present study had certain limitations. Due to the relatively small number of UC patients with opportunistic infection, we collected data for 5 years, which may have statistical biases, and the results need to be further confirmed by expanding the sample size and additional research is required to confirm whether our results can be generalized to other diseases associated with EBV and/or CMV infection populations. Second, residual confounding effects could not be excluded. We constructed multivariable logistic regression models and performed subgroup analyses to control for the effects of potential confounders on the relationship between NK cells and the EBV and/or CMV infection. Third, because this was a cross-sectional study, the causality of the relationship between the inflammatory potential of NK cells and the EBV and/or CMV infection could not be determined. Therefore, longitudinal studies are required to determine whether the observed relationship between NK cells and the EBV and/or CMV infection is causal.

## 5 Conclusion

There was a non-linear relationship between NK cells and EBV and/or CMV infection. In particular, the risk for EBV and/or CMV infection was not increased with increasing NK cells in individuals with NK cells≥10.169%, whereas the risk for EBV and/or CMV infection was increased with an decreasing NK cells in those with NK cells <10.169%. The risk of EBV and/or CMV infection increases only when NK cells are below a certain level.

## Data Availability

The datasets presented in this study can be found in online repositories. The names of the repository/repositories and accession number(s) can be found in the article/Supplementary material.

## Footnotes

^1^ http://www.Rproject.org
