# Correction: Relationship between the risk of intestinal mucosal Epstein–Barr virus and/or cytomegalovirus infection and peripheral blood NK cells numbers in patients with ulcerative colitis: a cross-sectional study in Chinese population

**DOI:** 10.3389/fmicb.2026.2023130

**Published:** 2026-10-26

**Authors:** Ye Tian, Jinghua Dai, Yunfeng Yang, Xiaofeng Guo, Wei Wang, Fengxia Li, Juzi Wang, Ruiyun Liu

**Affiliations:** 1 Department of Gastroenterology, Shanxi Provincial People's Hospital Affiliated to Shanxi Medical University, National Clinical Research Center for Digestive Diseases, Shanxi Inflammatory Bowel Disease Center, Taiyuan, China; 2 School of Nursing, Shanxi Medical University, Shanxi Provincial People's Hospital Affiliated to Shanxi Medical University, Taiyuan, China; 3 Department of Laboratory Medicine, Shanxi Provincial People's Hospital Affiliated to Shanxi Medical University, Taiyuan, China; 4 Nursing Department, Shanxi Provincial People's Hospital Affiliated to Shanxi Medical University, Taiyuan, China; 5 Shanxi Children's Hospital Affiliated to Shanxi Medical University, Taiyuan, China

**Keywords:** ulcerative colitis, cytomegalovirus, Epstein–Barr virus, opportunistic infections, NK cells

Affiliation “Shanxi Provincial People's Hospital *Affiliated to Shanxi Medical University*” was omitted from the paper. This affiliation has now been added for author(s) [Ye Tian, Jinghua Dai, Yunfeng Yang, Xiaofeng Guo, Wei Wang, Fengxia Li and Juzi Wang]. The correct affiliation should be read as follows: “^1^Department of Gastroenterology, Shanxi Provincial People's Hospital *Affiliated to Shanxi Medical University*, National Clinical Research Center for Digestive Diseases, Shanxi Inflammatory Bowel Disease Center, Taiyuan, China, ^2^School of Nursing, Shanxi Medical University, Shanxi Provincial People's Hospital *Affiliated to Shanxi Medical University*, Taiyuan, China, ^3^Department of Laboratory Medicine, Shanxi Provincial People's Hospital *Affiliated to Shanxi Medical University*, Taiyuan, China, ^4^Nursing Department, Shanxi Provincial People's Hospital *Affiliated to Shanxi Medical University*, Taiyuan, China, ^5^Shanxi Children's Hospital Affiliated to Shanxi Medical University, Taiyuan, China”

The original version of this article has beenupdated.

